# Genomic alterations in rectal tumors and response to neoadjuvant chemoradiotherapy: an exploratory study

**DOI:** 10.1186/1748-717X-6-161

**Published:** 2011-11-18

**Authors:** Chiara Molinari, Michela Ballardini, Nazario Teodorani, Massimo Giannini, Wainer Zoli, Ermanno Emiliani, Enrico Lucci, Alessandro Passardi, Paola Rosetti, Luca Saragoni, Massimo Guidoboni, Dino Amadori, Daniele Calistri

**Affiliations:** 1 Biosciences Laboratories, Istituto Scientifico Romagnolo per lo Studio e la Cura dei Tumori (I.R.S.T.), Meldola, Italy; 2 Unit of Biostatistics and Clinical Trials, I.R.S.T., Meldola, Italy; 3 Radiotherapy Unit, Santa Maria delle Croci Hospital, Ravenna, Italy; 4 Radiotherapy Unit, I.R.S.T., Meldola, Italy; 5 Department of General Surgery, Morgagni-Pierantoni Hospital, Forlì, Italy; 6 Department of Medical Oncology, I.R.S.T., Meldola, Italy; 7 Pathology Unit, Morgagni-Pierantoni Hospital, Forlì, Italy; 8 Immunotherapy and Cell Therapy Laboratory, I.R.S.T., Meldola, Italy

**Keywords:** Genomic alterations, rectal cancer, neoadjuvant chemoradiotherapy, ArrayCGH

## Abstract

**Background:**

Neoadjuvant chemoradiotherapy is the treatment of choice in advanced rectal cancer, even though there are many patients who will not benefit from it. There are still no effective methods for predicting which patients will respond or not. The present study aimed to define the genomic profile of rectal tumors and to identify alterations that are predictive of response in order to optimize therapeutic strategies.

**Methods:**

Forty-eight candidates for neoadjuvant chemoradiotherapy were recruited and their pretherapy biopsies analyzed by array Comparative Genomic Hybridization (aCGH). Pathologic response was evaluated by tumor regression grade.

**Results:**

Both Hidden Markov Model and Smoothing approaches identified similar alterations, with a prevalence of DNA gains. Non responsive patients had a different alteration profile from responsive ones, with a higher number of genome changes mainly located on 2q21, 3q29, 7p22-21, 7q21, 7q36, 8q23-24, 10p14-13, 13q12, 13q31-34, 16p13, 17p13-12 and 18q23 chromosomal regions.

**Conclusions:**

This exploratory study suggests that an in depth characterization of chromosomal alterations by aCGH would provide useful predictive information on response to neoadjuvant chemoradiotherapy and could help to optimize therapy in rectal cancer patients.

The data discussed in this study are available on the NCBI Gene Expression Omnibus [GEO: GSE25885].

## Background

The benefits of neoadjuvant chemoradiotherapy (NCRT) in rectal cancer are well documented. In particular, preoperative treatment is indicated to downsize tumors in order to achieve tumor-free margins, reduce tumor burden and increase the possibility of conservative surgery, which results in a high rate of sphincter preservation and significant improvement in local disease control and survival [1,2]. However, although complete pathologic response rates of 10-25% can be achieved, more than one third of patients either do not respond or show only modest response to treatment [3].

Whilst numerous studies have analyzed the correlation between expression levels of candidate genes and response to therapies [4,5], the predictive role of such genes is controversial and there is still no firm evidence upon which to base treatment strategies [6]. The gene expression profile evaluated by cDNA microarray has recently been found to provide indications about response of rectal tumors to NCRT [7-9], but such preliminary findings require confirmation in larger patient cohorts.

It is well known that the altered transcription of genes frequently depends on genomic copy number changes, such as deletion of one or both alleles of tumor suppressor genes, amplification of oncogenes or other rearrangements [10,11]. Although several basic research studies have highlighted the presence of non random patterns of DNA alterations in colorectal cancer [12-15], almost none of these alterations have been analyzed as predictive markers of response to clinical treatment, especially in rectal cancer. It was established only recently that genomic imbalances detected by metaphase Comparative Genomic Hybridization (CGH) could be of value for response prediction [16]. With respect to this technique, higher resolution mapping of chromosomal copy number changes can be achieved by array CGH (aCGH), a technique capably of accurately identifying even small variations in genomic DNA sequence [17,18].

The main objective of the present study was to define the molecular profile of rectal cancers in order to identify markers that are predictive of response to NCRT. The acquisition of more detailed genomic information would optimize treatment planning and lead to improved clinical and cost benefits.

## Methods

### Patients, samples and treatment

A series of 51 consecutive patients with a confirmed diagnosis of rectal adenocarcinoma localized in the mid-low rectum (up to 12 cm from the anal verge) and who were candidates for NCRT were considered eligible. The study was approved by the Local Ethics Committee, in accordance with the ethical standards laid down in the 1964 Declaration of Helsinki. All patients gave their written informed consent.

After pretherapeutic staging with a computerised tomography scan and also, in the majority of cases (> 80%), with endorectal ultrasonography, all patients were treated with a total dose of 50.4 Gy for 5-6 weeks with conventional fractionation. A daily dose of 225 mg/m^2 ^of 5-fluorouracil was infused by central catheter during radiotherapy. Surgery was planned 6-8 weeks after completion of chemoradiotherapy. The median duration of the interval between the day after the end of therapy and surgery was 52.3 ± 10.9 days (range 40-91). Compliance to treatment was good as only 6% (n = 3) of enrolled patients were excluded because of high toxicity.

Blood samples and tumor biopsies were collected from patients before therapy. Two biopsies from tumor areas were taken from each patient; the first was used to obtain histopathologic confirmation of tumor diagnosis, while the second was immediately stored at -80°C and, after microscopic verification of the presence of > 70% of tumor cells in the former, used for genomic profile determination. The pathologic response to NCRT was evaluated using the tumor regression grade (TRG) classification, according to the criteria proposed by Dworak [19].

### Immunohistochemistry

In parallel, two conventional markers of proliferative (Ki67) and apoptotic (p21^WAF1^) processes were determined by immunohistochemical (IHC) methods using the following primary antibodies: anti-Ki67 (clone MM1; Leica Microsystems, Heidelberg, Germany; working dilution 1:100) and anti-p21^WAF1 ^(clone DC-60.2; Neomarkers, Fremont, CA, USA; working dilution 1:50). For antigen retrieval, sections were treated with 10 mM citrate (pH 6.0) at 98°C for 40 min and were then immunostained with LSAB+ System-HRP Kit (Dako, Carpinteria, CA, USA) according to the manufacturer's specifications. For Ki67, all washes were performed with TBS rather than PBS. Both antibodies were visualized by diaminobenzidine. Two independent observers with no prior knowledge of clinicopathologic data performed blinded immunohistochemical analysis. At least 500 cells were evaluated in representative microscopic fields and results were expressed as a percentage of cell showing nuclear Ki67 or p21^WAF1 ^staining.

### Array comparative genomic hybridization (aCGH)

Whole genome CGH arrays (Cytochip, Bluegnome, Cambridge, UK), which cover the entire human genome at a 1-Mb resolution and, in subtelomeric regions, at a median 250-Kb resolution, were used for the analysis. Each clone in the array was spotted in quadruplicate. Genomic DNA was isolated from tumor tissue using QIAamp DNA MiniKit (Qiagen, Hilden, Germany). A pool of normal female or male genomic DNA from healthy individuals was used as a reference, depending on patient gender. Approximately 1.2 μg of DNA was labelled by random priming (BioPrime Labeling System, Invitrogen, Milan, Italy), using 1.4 μl of 1 mmol/L Cy3-dCTP or Cy5-dCTP (Perkin-Elmer Life Sciences, Waltham, MA, USA) according to the manufacturer's instructions. Equal amounts of labelled tumor and reference DNA were mixed and unincorporated fluorochromes were removed using Bioprime Array-CGH Purification Module (Invitrogen). DNA was then precipitated with 140 μl of blocking mix (TechnoGenetics-Bouty, Milan, Italy), 6 μl of yeast tRNA 10 mg/ml (Sigma-Aldrich, St. Louis, MO, USA), 0.1 V/V of 3 M sodium acetate pH 5.2 and 2 V/V of absolute ethanol. The DNA pellet was dissolved in 10 μl of distilled water and 30 μl of hybridization solution (TechnoGenetics-Bouty). After denaturation at 72°C for 10 min and incubation at 37°C for 30 min, the solution was applied to the array. Hybridization was done in a Hyb Chamber (Biorad, Hercules, CA, USA), which was then incubated at 42°C for 40 h. Finally, the slides were washed according to the supplier's instructions, dried by spinning in a centrifuge for 5 min at 1000 rpm and scanned on a VersaArray ChipReader scanner (Biorad).

### Image and data analysis

Images were analyzed using BlueFuse version 3.5 software (BlueGnome), permitting an automated approach to aCGH. Spots were excluded when the quality flag was < 1, the confidence value < 0.3 or the standard deviation of quadruplicate > 0.2. Log_2 _ratios of spots that were not excluded were normalized using block median or block lowess approaches. Alteration analysis was performed using two different approaches: (1) aCGH-Smooth approach [20], implemented in BlueFuse version 3.5 software; (2) Hidden Markov Model (HMM) approach, implemented in the aCGH package [21-23]. Molecular analyses were performed in a blinded manner.

### Statistics

Statistical analyses were carried out using SPSS (SPSS Inc, Chicago, IL, USA) and SAS (release 9.1, SAS Institute, Cary, NC, USA) statistical software. The Kruskal-Wallis non parametric test was used to assess the predictive relevance of genomic instability, expressed as the fraction of genome altered (FGA) in relation to response to therapy in different stage and age subgroups. The Pearson chi-square test and Fisher's exact test were used to analyze the relation between patient characteristics and response to NCRT, defined according to TRG criteria, and the difference in the frequency of altered regions among response groups, respectively.

## Results

### Clinical parameters and pathologic response to NCRT

Information on patient and tumor characteristics and on response to NCRT is shown in Table 1. On the basis of TRG criteria proposed by Dworak, 13 (27%) patients reached TRG4, *ie*, complete tumor regression; 8 (17%), 17 (35%) and 9 (19%) reached TRG3, TRG2, and TRG1, respectively. Only 1 (2%) case was classified as TRG0, *ie*, did not show any pathologic response. To further simplify the analysis and summarize clinically relevant results, patients were grouped into two subsets, as already done by other authors [7]: those who achieved TRG0-2 (56%) were defined as non responders, while patients who obtained TRG3-4 (44%) were considered responders. Analysis of the relationship between clinical pretreatment parameters and pathologic response did not reveal any significant association. However, a significant correlation (*p *= 0.0004) was found between TRG and ypT, with 57% of responsive patients who were ypT0 and 52% of non responsive patients who were ypT3. Conversely, no association was found between ypN and TRG.

**Table 1:** Patient and tumor characteristics

	No. of patients	(%)
**Gender**		
Male	36	(75)
Female	12	(25)
**Age (y)**		
Median	66	
Range	37-82	
**uT stage**		
2	7	(15)
3	39	(81)
4	2	(4)
**uN stage**		
0	27	(56)
+	21	(44)
ypT stage*		
0	14	(30)
1	6	(13)
2	11	(23)
3	16	(34)
ypN stage*		
0	37	(78)
+	10	(22)
**TRG**^§^		
0	1	(2)
1	9	(19)
2	17	(35)
3	8	(17)
4	13	(27)
**TRG response**		
Responsive	21	(44)
Non Responsive	27	(56)

*Data was not available for one patient

^§ ^According to Dworak [19]

### Ki67 and p21^WAF1 ^expression and pathologic response to NCRT

Ki67 and p21^WAF1 ^expression was determined in 35/48 (73%) and 34/48 (71%) of cases, respectively, due to insufficient bioptic material. Median Ki67 index was 69% in the overall series (range 23-93) and was similar in responsive (69%; range 23-85) and non-responsive (69.5%; range 29-93) patients. With regard to p21^WAF1^, the median value of positive cells was 4% (range 0-46), specifically 5.5% (range 0-17) in the responsive group and 2.5% (range 0-46) in the non responsive group. No correlations were observed between pathologic response and Ki67 or p21^WAF1 ^expression.

### Genomic profile and pathologic response to NCRT

#### Validation of aCGH approach

The performance and reproducibility of our aCGH approach was validated by dye swap and normal/normal experiments (data not shown). Specifically, we performed three array CGH analyses of normal female (XX) DNA against normal male (XY) DNA extracted from blood samples. No copy number changes were detected apart from sexual chromosomes. As expected, the log_2 _values of the average normalized fluorescence ratios for all the autosomal loci showed two copies, whereas the fluorescence ratios for the chromosomes X and Y loci indicated the gain and loss of one DNA copy, respectively. These experiments also permitted us to establish the best thresholds for significant DNA alterations (loss: log_2 _ratios < - 0.3219; gain: log_2 _ratios > 0.2630).

#### HMM analysis and unsupervised hierarchical clustering

The FGA identified by HMM analysis was 4.19% ± 7% (range 1.7-29%), with FGA-gain of 2.4% ± 4.4% (range 0.47-18.1%) and FGA-loss of 1.8% ± 3.2% (range 0.2 -15.6%). No significant associations were observed between FGA and pathologic response, other clinical parameters, or Ki67/p21^WAF1 ^expression.

**Figure 1:**
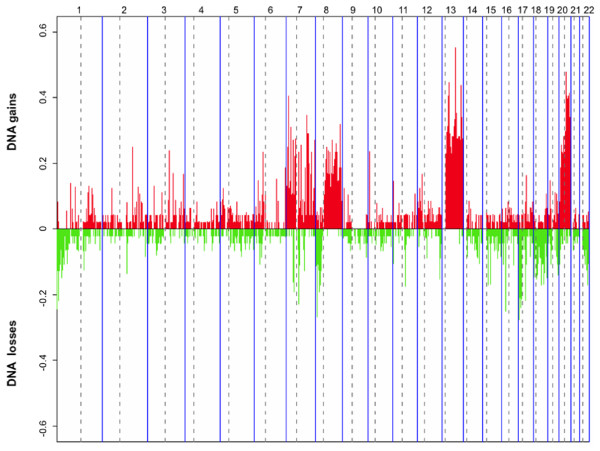
**Frequencies of all significant gains and losses in 48 rectal cancers, obtained with HMM analysis**. Red bars show copy number gains and green bars, copy number losses. The boundaries of individual chromosomes and the location of centromers are indicated by vertical bars.

The loss of genomic copies was more frequent on chromosome arms 1p, 8p, 17p, 18p and 18q, and 22q, while chromosome regions on 7p and 7q, 8q,13q, and 20p and 20q were frequently amplified (Figure 1). Small regions of relevant copy number changes (log_2 _ratio > 0.9 for high-level gain and log_2 _ratio < -0.75 for high-magnitude deletions) were also observed. High-level gain was observed in 10 tumors, 9 of which were non responsive according to TRG classification, and occurred most frequently in chromosome 7 (4/10), 13 (4/10), 17 (3/10) and 20 (5/10).

**Figure 2:**
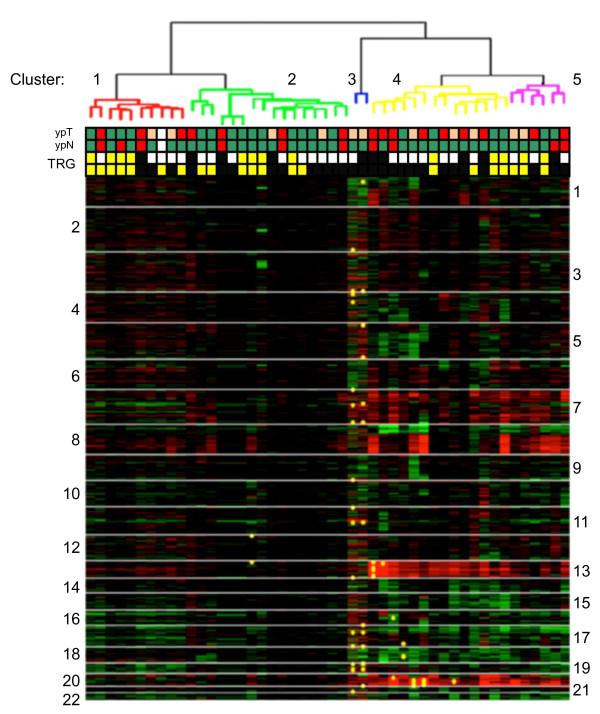
**Unsupervised hierarchical clustering of 48 rectal cancers analyzed by HMM**. Clones are shown in chromosomal order from 1ptel to 22qtel (red, gain; green, loss; yellow spots, high level gain). Tumor regression grade (TRG) classification: responders (TRG3-4) yellow; non responders (TRG0-2) black. Extreme response classes are reported in the upper line: TRG4 yellow and TRG0-1 black. ypT status: ypT0-1green; ypT2 orange and ypT3 red. ypN status: ypN0 green, ypN1 red and ypNx white.

Unsupervised hierarchical clustering of the 48 tumors showed molecular heterogeneity and 5 well-defined clusters (1-5) were identified (Figure 2). The noteworthy peculiarity among the clusters was the difference in response to NCRT. The three main clusters (1, 2 and 4) included 83% of cases. In cluster 1 (21% of cases), 70% of patients were responders on the basis of TRG, while in clusters 2 (33%) and 4 (29%), 43% and 29% of patients responded to NCRT, respectively (*p <*0.0001). If only patients with extreme differences in response to NCRT (TRG4 versus TRG0-1) were considered, for clusters 1, 2 and 4 the total number of patients that reached TRG4 were 40%, 25% and 21%, respectively (*p *= 0.0005). We also performed supervised clustering but did not identify any specific signature, possibly because of the small number of cases (data not shown).

### Smoothing analysis

Smoothing analysis of all DNA copy number changes showed that the FGA was 1.33% ± 4% (range 0-14.6%). The most frequent alteration was the gain of genomic regions. In our tumor series, a total of 3888 clones were gained (FGA-gain 1.17% ± 3.7%; range 0-14.6%) and 517 were lost (FGA-loss 0.16% ± 0.8%; range 0-3%). Some alterations involved entire or extensive portions of chromosome arms and mainly affected the long arm of chromosomes 13 and 20, with a 31% and 38% gain frequency, respectively. Other common alterations were detected on specific genomic regions, *e.g*. on 7p22-21 and 8q12, which were amplified in about 13% and 16% of patients, respectively. An average of 80 clones showed high-level alterations, mainly gain, with a high percentage (49%) located on chromosome 20q.

The analysis of the relationship between FGA and response to NCRT according to TRG did not reach statistical significance. However, the evaluation of tumor aCGH profiles clearly showed differences between the two response groups with regard to type and/or frequency of DNA alterations at specific chromosomal regions (2q21, 3q29, 7p22-21, 7q21, 7q36, 8q23-24, 10p14-13, 13q12, 13q31-34, 16p13, 17p13-12, 18q23) (Table 2). Among the most altered clones were those mapping in 2q21.1, which were amplified in non responsive patients and deleted in responsive ones. A number of clones were altered in only responsive or non responsive patients, such as clones in 7q21.11, amplified in 10% of responsive patients, or clones in 7q36.1-3 or 16p13.3 regions, amplified in 15% and 11% of non responsive patients, respectively. Finally, high-level alterations were distributed differently with respect to response to NCRT, with 85% of patients who showed this kind of alteration in the group of non responsive cases.

**Table 2:** Number and frequency of principal aberrations distinguishing patients belonging to extreme response classes

Chromosomal band	TRG0-1		TRG4		*p *value
			
	Amplifications	Deletions	Amplifications	Deletions	
	%	%	%	%	
2q21	10	0	0	15	< 0.0001
3q29	20	0	0	0	< 0.0001
7p22-21	20	0	8	0	0.0237
7q21	0	0	15	0	< 0.0001
7q36	20	0	8	0	0.0237
8q23-24	30	0	15	0	0.0171
10p14-13	20	0	0	0	< 0.0001
13q12	30	0	15	0	0.0171
13q31-32	30	0	15	0	0.0171
13q34	40	0	15	0	< 0.0001
16p13	20	0	0	0	< 0.0001
17p13-12	0	20	0	8	0.0237
18q23	10	20	0	0	< 0.0001

### Genomic profile and pathologic TNM after NCRT

Information on pathologic stage and lymph node status after NCRT (ypT and ypN) was available for 47/48 patients. Among these, 30% had ypT0 while 13%, 23% and 34% had ypT1, ypT2, ypT3 respectively. With regard to ypN, 78% of patients were ypN0, whereas 22% had a lymph node involvement at the time of surgery.

FGA was significantly different in tumors that showed different pathologic stages after NCRT, with a higher frequency of alterations in tumors that had an advanced T stage (*p *< 0.05). With respect to ypT stage, the five groups identified by hierarchical clustering showed a different frequency of ypT0-1, ypT2 and ypT3 tumors. In particular, grouping together clusters 1 and 2 (left side of Figure 2), and clusters 3, 4 and 5 (right side of Figure 2), the percentage of different ypT-stage tumors was as follows: 56% and 27% of ypT0-1, 16% and 32% of ypT2, and 28% and 41% of ypT3, respectively. This difference was statistically significant (*p <*0.0001). No association was observed between FGA and ypN, and no differences were noted between clusters with regard to ypN status.

## Discussion

Neoadjuvant chemoradiotherapy followed by surgery is accepted as the therapy of choice in rectal cancer. However, although good results have been obtained with current treatment strategies, clinically identical tumors sometimes differ in their response, and more personalized treatments would undoubtedly yield greater benefits [24].

The two most widely adopted systems of assessing response are tumor regression grade (TRG), according to Mandard or Dworak criteria, and downstaging. However, there is still no general consensus as to which one is the most accurate and the most clinically relevant for patient prognosis [7,25-28]. In our study, we adopted the TRG system proposed by Dworak [19] (the response classification system used by our pathologists), grouping TRG 0-2 tumors as non responders and TRG 3-4 tumors as responders. This type of division has also been proposed by numerous other authors [19,7,25] and has been acknowledged as having a prognostic impact [25].

We did not find any significant correlation between pathologic response to NCRT and clinical pretreatment parameters. In addition, the small number of patients evaluated prevents us from drawing any definitive conclusions about the correlation between Ki67 and p21^WAF1 ^expression (markers involved in cell cycle control and proliferation) and TRG. We did, however, observe a higher median value of p21^WAF1 ^protein expression in responsive tumors.

Although several studies have investigated colorectal cancer by the aCGH approach to better characterize the alterations of this tumor [29-31], few have focused on rectal cancer [32]. Furthermore, to our knowledge, the only study analyzing rectal cancer genomic imbalances in relation to response used metaphase CGH and concluded that the probability of detecting copy number changes by chance was high [16]. In our case series, analysis of tumor DNA using higher resolution scanning with aCGH showed that the fraction of altered genome obtained using the HMM approach (4.19%) was slightly higher than that obtained by Smoothing analysis (1.33%); this is to be expected as the latter considers only larger alterations. However, both approaches appear to identify similar alterations, with a prevalence of DNA gains.

According to the literature [12-14,16,29-32], the most frequent DNA alterations specific for this tumor were gains of chromosome arms 7p and 7q, 8q, 13q, 20p and 20q, and losses in 1p, 8p, 17p, 18p and 18q and 22q. In our case-series, 38% of tumors showed a gain at 20q chromosome arm, where multiple candidate oncogenes associated with increased proliferative activity, reduced survival and progression of colorectal cancer (*e.g*. ZNF217, CYP24 and AURKA), are mapped [12,33].

With regard to differences in genomic alterations between responsive and non responsive tumors, whether revealed by HMM or Smoothing analyses, our results showed that a higher number of genome changes was associated, albeit not significantly, with resistance to treatment, in contrast to findings published by Grade and colleagues [16].

The overall difference in genomic instability between responders and non responders could be due to the fact that gene drivers of genetic alterations, *i.e*. genes involved in DNA repair, mitotic checkpoints and carcinogen detoxification, are frequently also involved in the response to therapies which act by targeting proliferating cells and damaging DNA. In our case series, however, the association between FGA and pathologic response was not significant, probably owing to the small number of patients enrolled and to the complex pathways involved in genomic stability which may themselves influence response to therapy.

Our clustering analysis indicates a difference in response to NCRT among patients with a specific DNA alteration pattern. In particular, in cluster 1 we observed 70% of responders, whereas in cluster 4 more than 70% of patients were non responders. Even considering only the extreme response classes (TRG4 as responders versus TRG0-1 as non responders), there is a similar distribution of TRG4 in clusters 1, 2 and 4. Moreover, in view of the important role played by final pathologic stage in determining prognosis, we also took into account the relationships between ypTNM, response to therapy and genomic alterations. A significant correlation was found between TRG and ypT, as well as a significant association between HMM-detected FGA and ypT stage. In fact, a higher number of DNA alterations in pretreatment tumors was associated with higher pathologic tumor stage obtained after NCRT. In the hierarchical clustering was confirmed a statistically different frequency of ypT: in particular, in clusters 1 and 2 (46% of non responders), the vast majority of cases were ypT0-T1, whereas in clusters 3-5 (68% of non responders), ypT3 tumors were predominant (*p <*0.0001).

In our case series, specific chromosomal regions presenting alterations capable of distinguishing between responsive and non responsive tumors, *e.g*. 2q21, 3q29, 7p22-21, 7q21, 7q36, 8q23-24, 10p14-13, 13q12, 13q31-34, 16p13, 17p13-12 and 18q23 were observed. Several alterations, such as that in the 7q36.1-7q36.3 region (sometimes amplified in non responsive patients), appear interesting as a number of important genes map there; these genes are mainly involved in transcription, cell-cell signalling, chromosome stability and DNA repair, cell growth, differentiation and oncogenic transformation, *e.g*. MLL3 and XRCC2, known to be altered in colorectal cancer [34,35]. We also observed fairly marked differences in chromosome 13 alterations between tumors from patients who benefited from NCRT and those from patients who did not. In fact, both HMM and Smoothing approaches identified a gain of several clones spanning the 13q31-34 region in numerous patients, the vast majority of whom were not responsive to treatment.

The preferential gain of 13q regions in non responders is of particular interest because of the relevance of a number of genes located within these regions, *e.g*. Wave3, which contributes to tumor cell invasion and metastasis [36]; XPO4, an exportin that plays a primary role in the nucleocytoplasmic shuttling of Smad3 [37]; and APC6/CDC16, a subunit of the anaphase-promoting complex in which amplification and overexpression may lead to chromosomal instability [38].

Furthermore, genes that play a role in the control of cell division and growth regulation, migration and chromosomal instability (GPC5, GPC6, HS6ST3) map in these chromosomal bands [39,40]. Two other interesting genes, CLDN10 and ABCC4, localized in 13q31-34, may be involved in determining resistance to therapy. The former encodes a member of the claudin family, integral membrane proteins and components of tight junction strands associated with cell invasion and migration [41]. The latter gene, ABCC4/MRP4, belongs to the ABC-type multidrug transporter family that mediates drug resistance by energy-dependent drug efflux from cells and seems to be implicated in resistance to purine analogs and to other nucleoside-based antiviral drugs [42,43].

Another interesting region, deleted in some non responsive patients of our case series, is localized at 17p12.1. LLGL1, a candidate tumor suppressor gene involved in the maintenance of epithelial integrity through its cytoskeletal interactions, maps in this chromosomal band. As highlighted in some studies, LLGL1 is deleted preferentially in CIN-type tumors [30], and the downregulation of this gene would seem to contribute to colorectal cancer progression [44].

It is known that there is often a correlation between genomic copy number and gene expression levels as DNA imbalances seem to have a direct effect on the deregulation of the transcriptional profile of cancer cells [45]. Gene expression signature studies in rectal cancer patients subjected to neoadjuvant therapy have shown that different approaches yield different gene sets which may be predictive of response. Genes involved in common molecular pathways and cellular processes have been identified, including those involved in DNA damage repair, microtubule organization, apoptosis, transcription, cell growth, signal transduction, drug metabolism, and transport functions [7-9,46]. Our results are in line with such findings as the main differences in DNA copy number between responsive and non responsive patients were observed in genes involved in DNA damage response, transcription, cell cycle and mitosis regulation, excretion, intercellular junction assembly and calcium-independent cell-cell adhesion pathways.

## Conclusions

The results from the present study indicate that the aCGH technique could be a potentially useful tool to better characterize rectal cancer. Our findings also suggest that genome-wide profiling could be used to distinguish between patients who respond to NCRT and those who do not, although further genomic studies in larger patient cohorts are needed to better classify the differences in DNA copy number changes between responsive and non responsive patients. Research is ongoing to confirm these preliminary data.

## List of abbreviations

NCRT: neoadjuvant chemoradiotherapy; aCGH: array comparative genomic hybridization; TRG: tumor regression grade; HMM: Hidden Markov Model; FGA: fraction of genome altered.

## Competing interests

The authors declare that they have no competing interests.

## Authors' contributions

In keeping with the latest guidelines of the International Committee of Medical Journal Editors, each author's contribution to the paper has been quantified as follows: CM carried out the molecular studies, participated in the statistical analysis and drafted the manuscript; MB performed the statistical analysis; NT participated in the study design and in the enrollment of patients; MG partecipated in the enrollment of patients; WZ participated in the study design and in revising the manuscript; EE partecipated in the study design and in the enrollment of patients; EL performed the collection of samples; AP and PR participated in the enrollment of patients; LS carried out the histopathologic confirmation of tumor diagnosis, the determination of the pathological response to therapy and collaborate to immunohistochemical analysis; MG performed immunohistochemical analysis; DA conceived the study; DC participated in the methodology development, in the study design and in drafting the manuscript. All authors read and approved the final manuscript.
